# Potential inhibitory effect of fish, maize, and whey protein hydrolysates on advanced glycation end‐products (AGEs)

**DOI:** 10.1002/fsn3.3289

**Published:** 2023-02-28

**Authors:** Faezeh Arasteh, Mohsen Barzegar, Hassan Ahmadi Gavlighi

**Affiliations:** ^1^ Department of Food Science and Technology, Faculty of Agriculture Tarbiat Modares University Tehran Iran

**Keywords:** advanced glycation end‐products, amino acids, antiglycation, bioactive peptides, hydrolysate

## Abstract

Advanced glycation end‐products (AGEs) are produced in the final stage of the Maillard reaction. AGEs formation may be inhibited by natural hydrolysates derived from plant or animal sources. The present study aimed to investigate the antiglycation potential of fish, maize, and whey protein hydrolysates. It was carried out in four model systems, Bovine serum albumin (BSA)‐Glucose, BSA‐Fructose, BSA‐Sorbitol, and BSA‐HFCS (high fructose corn syrup), by evaluation of fluorescent intensity of AGEs after seven days of reaction at 37°C. The results showed that the highest inhibitory effect belonged to 0.16% of FPH (fish protein hydrolysate, percent inhibition ~99.0%), whereas maize protein hydrolysate (MPH) had lower antiglycation activity in comparison with FPH. Among all hydrolysates, whey protein hydrolysate with the lowest degree of hydrolysis showed the weakest inhibitory activity. Overall, our results indicated that the investigated hydrolysates, particularly FPH, have promising antiglycation potential and can be recommended for the production of functional foods.

## Graphical abstract

Advanced glycation end‐products (AGEs) are produced in the food processing and they have some side effects on consumers. In this study, we used three natural hydrolysates of fish, maize, and whey protein as antiglycation agents. It was carried out in four model systems, Bovine serum albumin (BSA)‐Glucose, BSA‐Fructose, BSA‐Sorbitol, and BSA‐HFCS (high fructose corn syrup). Our results indicated that the investigated hydrolysates, particularly FPH, have promising antiglycation potential and can be recommended for the production of functional foods.

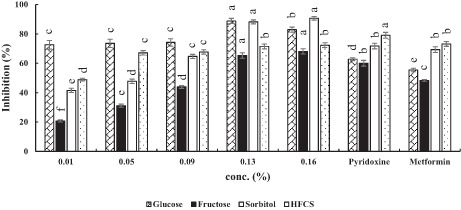

## 1 INTRODUCTION

One of the important reactions that occurs during food processing is known as glycation (Zhou et al., 2020). This reaction happens between amine groups of proteins, nucleic acids, or amino acids and hydroxyl groups of reducing sugars. The most dangerous chemicals are gradually formed during this reaction via a variety of distinctive and complicated Maillard pathways known as advanced glycation end‐products (AGEs). On the other hand, it can provide several sensorial properties, for instance, flavor, color, and taste, in a wide range of foodstuffs; also it might be unfavorable by keeping the process progress to the last stages. So, food producers attempted to reduce this reaction during different processing.

Hence, one viable approach to overcome these problems is using AGE inhibitors. Synthetic chemical compounds such as piroxicam and its derivatives (Ullah et al., 2021), metformin (Adeshara et al., 2020), glipizide (Adeshara & Tupe, 2016), colchicine, mefenamic acid, as well as meloxicam (Rasheed et al., 2018) have been tested as antiglycation and antioxidant agents under different conditions whether in vitro or in vivo. Due to the numerous side effects of synthetic inhibitors, specifically their toxicity, natural components seem to be safer for preventing the glycation. Recently, there has been increased interest in using natural compounds produced from different sources to replace chemical inhibitors.

The antiglycation potential of olive mill wastewater polyphenol powders (OMWP) was tested in cookies model system and proved to be a potential inhibitor for Amadori products and dicarbonyl compound's formation (Troise et al., 2020). Another in vitro study investigated the antiglycation activity of various extracts of common spices and plant extracts, namely, allspice, different types of peppers, thyme leaves, etc. (Favre et al., 2022). Antiglycation potential of other natural components, namely, green tea extract (Poojary et al., 2020), *cympobogon citratus* (lemon; Kadu & Pantawane, 2019), grape pomace (Harsha & Lavelli, 2019), *stevia rebaudiana* (Ali et al., 2022), citrus wastes (Fernandes et al., 2020), and hazelnut skin (Spanguolo et al., 2021), has been extensively studied.

The antiglycation activity of different peptides extracted from soya bean (Kuerban et al., 2017), *Vicia faba* (Kuerban, Al‐Gharafi, et al., 2020), *Lens culinaris* (Kuerban, Al‐Malki, et al., 2020), and barely, walnut, almond, etc. (Kani et al., 2019) has been studied by other researchers.

Fish, maize, and whey are protein‐rich foodstuffs consumed on a large scale and applied as supplements because of their plenty of bioactivity potentials. For instance, in a study conducted by Idowu et al. (2019), fish protein hydrolysates represented antioxidant, antimicrobial, antitumor, and ACE inhibiting activities. In addition, biological functions of both maize and whey peptides, such as antioxidant, antitumor, and immunomodulatory activity, were investigated as well (Li et al., 2013).

Although research studies have reported some biological activities of fish, maize, and whey peptides (Idowu et al., 2021; Ma et al., 2014; Zhaung et al., 2013), there is still a lack of information about antiglycation activities of mentioned peptides under in vitro conditions. Therefore, the purpose of the present study was to determine the antiglycation activities of three selected peptides under in vitro conditions.

## 2 MATERIALS AND METHODS

### 2.1 Material

d‐sorbitol, d‐glucose, d‐fructose, Bovine serum albumin (BSA), and sodium azide were purchased from Merck Chemical Co. High fructose corn syrup (with 55:45 fructose‐to‐glucose ratio) was obtained from Zar Fructose Company. Pyridoxine hydrochloride (B6 vitamin) and metformin hydrochloride were provided by Alborz Darou Co. Maize, fish, and whey protein hydrolysates, with 17.0%, 22.0%, and 14.0% degree of hydrolysis (DH), had been already produced in our laboratory (amino acids profiles of all three peptides are shown in Table 1). All other chemicals were obtained from Merck Chemical Co. and Sigma‐Aldrich Chemicals Co. with the highest purity available.

**TABLE 1:** Amino acids profiles of the three studied hydrolysates (g of amino acid/100 g of hydrolysate).

Hydrophilic amino acid				Hydrophobic amino acid			
	MPH	FPH	WPH		MPH	FPH	WPH
Aspartic acid + asparagine	4.69	6.76	10.91	Alanine	4.89	7.05	4.90
Glutamic acid + glutamine	22.01	11.61	17.65	Proline	5.80	3.22	5.45
Serine	13.74	4.82	5.02	Valine	6.30	5.66	5.01
Glycine	17.86	5.60	1.89	Methionine	0.51	4.25	2.18
Histidine	1.23	4.59	2.20	Isoleucine	2.21	4.40	4.82
Arginine	2.02	7.01	2.79	Leucine	8.40	8.83	11.07
Threonine	0.28	5.21	5.30	Phenylalanine	2.16	6.15	3.39
Cysteine	0.48	0.46	2.42	Tryptophan	ND	0.87	1.77
Tyrosine	3.01	5.32	3.46	Total content	30.27	40.43	38.59
Lysine	4.41	8.19	9.77				
Total content	69.73	59.57	61.41				

Abbreviation: ND, not detected.

### 2.2 Preliminary experiment

In order to find the best concentration range of each hydrolysate with the proper antiglycation activity, a wide range of concentrations (0.1%–0.7% of hydrolysate) were examined (Data not shown). Based on the results, the best activity range for maize, fish, and whey hydrolysates was between 0.01% and 0.16% and selected for further experiments.

### 2.3 Determination of antiglycation activity

The antiglycation activity of all three hydrolysates was evaluated by the method described by Kuerban et al. (2017) with slight modifications. Briefly, BSA 0.5 mL (1.00%) was prepared in PBS, pH = 7.4 with sodium azide (0.002% w/w) to inhibit microbial growth. Then, 0.5 mL of fructose, glucose, sorbitol, and HFCS solution (0.4 mM) was separately added to BSA solution. Afterward, 0.1 mL peptides (0.01%, 0.05%, 0.06%, 0.09%, 0.11%, 0.13%, 0.16%, and 0.21%) was dissolved in PBS (pH 7.4) and added to the reaction solutions. The control sample contained all the reaction components without hydrolysates. Two standard compounds, namely, metformin hydrochloride (1.0 mM) and pyridoxine hydrochloride (B_6_ vitamin, 1.0 mM), were used as positive controls. All solutions were shaken gradually during the reaction time. Finally, solutions were incubated in a dark place at 37°C for 7 days. All experiments were performed in three replicates.

After incubation, 0.1 mL of the reaction mixture was added to the ELISA plate 384 wells to measure the amount of produced fluorescent AGEs using an Agilent fluorescence spectrometer (model Cytation 3) with excitation and emission wavelengths of 330 and 470 nm, respectively. The experiment was prepared in triplicate. The percentage of antiglycation activity was calculated using the following formula:

(1) $$\text{Inhibition}\%=\left\{1-\left[\left(F_{t}–F_{b}\right)/\left(F_{c}–F_{b}\right)\right]\right\}\times 100$$

where *F*_*t*_, *F*_*b*_, and *F*_*c*_ are the fluorescence intensities of the test sample, blank, and control, respectively.

### 2.4 Statistical analysis

All experiments were performed in triplicate. Each obtained data was expressed as mean value ± standard deviation (*n* = 3). Statistical analysis of the mean values was performed by one‐way analysis of variance (ANOVA) using Mini table 16 Software (Minitab Inc.). *p* < .05 was considered as the statistically significant level.

## 3 RESULTS AND DISCUSSION

### 3.1 Determination of antiglycation activity of maize protein hydrolysates (MPH)

**FIGURE 1:**
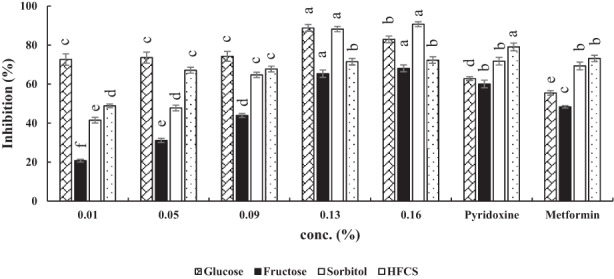
Antiglycation activities of maize protein hydrolysates in four model systems. Results are represented as mean ± SD (*n* = 3). Different letters on the columns indicate significant differences (*p* < .05). Concentration of metformin and pyridoxine was 1.0 mM.

Increasing the hydrolysate concentration up to 0.09% had no effect on the BSA‐Glucose system (Figure 1). However, by increasing the concentration to 0.13%, antiglycation activity increased to 88.7%. There was no increase (~83%) at 0.16% level hydrolysate. However, the inhibitory activity of maize peptides at all concentrations was higher than pyridoxine and metformin. It may be because of higher functional groups in maize peptides in comparison with those of standards (Sarmah & Roy, 2021).

By increasing the hydrolysate concentration in the BSA‐Fructose system, antiglycation activity reached 68%, and at concentrations more than 0.90%, MPH activity was significantly (*p* < .05) greater than pyridoxine (60.05%) and metformin (48.35%).

The lowest antiglycation activity was observed in the BSA‐Sorbitol system at 0.01% of natural inhibitor (MPH, 40%), while its efficacy reached 90% at 0.16%. In other words, MPH significantly inhibited AGE formation at higher concentrations (*p* < .05). However, the inhibitory activities of pyridoxine and metformin were 71.74% and 69.29%, respectively. In this system, the same as BSA‐Fructose system, at concentration higher than 0.09%, MPH's antiglycation activity was higher than both standards.

Regarding BSA‐HFCS model, MPH inhibitory activity was 48.85% at 0.01%. Then, by increasing the MPH concentration up to 0.16%, hydrolysate antiglycation activity reached 72%. In this system, both standards showed higher activity than MPH.

The reason for more inhibition at higher concentrations in some hydrolysates may be related to saturation of the readily available active groups of blockers (Mazumder et al., 2019). The treatment of MPH could gradually restrain AGE formation, showing a dose‐dependent manner (Deng, Wang, Zhang, Xie, & Huang, 2023; Jia et al., 2023).

### 3.2 Determination of antiglycation activity of FPH

**FIGURE 2:**
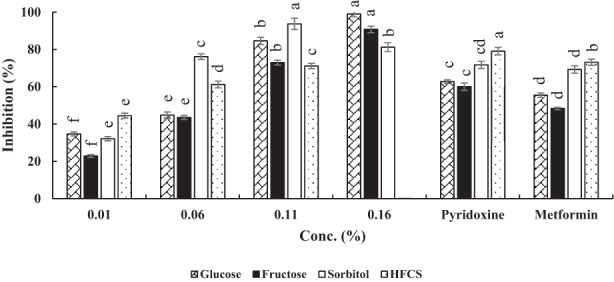
Antiglycation activities of fish protein hydrolysates in four model systems. Results are represented as mean ± SD (*n* = 3). Different letters on the columns indicate significant differences (*p* < .05). Concentration of metformin and pyridoxine was 1.0 mM.

The results of antiglycation activity of FPH are shown in Figure 2. As seen in Figure 2, percentage of inhibition in the BSA‐Glucose system was slightly less than 40% at beginning, whereas it increased significantly and reached nearly 100% at the highest concentration of FPH (0.16%; *p* < .05). The inhibitory activities of FPH at 0.11% and 0.16% were significantly higher than the pyridoxine (62.74%) and metformin (55.46%). In accordance with a study conducted by Deng, Wang, Zhang, Xie, and Huang (2023), bioactive peptides might show much stronger inhibitory effect compared with ferulic acid, a highly reactive phenolic compound, and cause AGE formation to have a downward trend.

In the BSA‐Fructose system, at 0.01%, the peptide's antiglycation activity was about 20%. The inhibitory effect of FPH on glycation reaction showed a dose‐dependent manner and the highest activity (90%) was observed at 0.16% of FPH that was significantly different from other concentrations (*p* < .05). Compared with pyridoxine and metformin, the inhibitory effects of the FPH at 0.11% and 0.16% were higher than both standards.

In the BSA‐Sorbitol system, the initial antiglycation activity was 32.1% (at 0.01%). A positive correlation was observed between the FPH concentration and antiglycation activity and reached 93.72% (at 0.11% of FPH). However, at 0.16% of FPH, the inhibitory activity decreased to ~80%. Also, in this system, the FPH's activity was more than pyridoxine and metformin.

Examining the BSA‐HFCS system revealed that at 0.01% FPH, antiglycation activity initiated at 44.43% and progressed to 71.15% at 0.11%. However, in this system, FPH showed no inhibitory effect at concentration of 0.16%. As seen in Figure 2, both synthetic standards have higher antiglycation activity than FPH. It is worth noting that some natural substances might lose their inhibitory potential at higher concentrations and therefore, act as pro‐oxidant agents and increase the glycation reaction progress (Rajaei et al., 2010; Sarmah & Roy, 2021). Based on the previous studies, bioactive compounds such as peptides can suppress protein aggregation by scavenging free radicals, which can facilitate oxidative damage and AGE formation (Deng, Wang, Zhang, Zhang, et al., 2023; Zhu et al., 2022).

### 3.3 Determination of antiglycation activity of WPH

**FIGURE 3:**
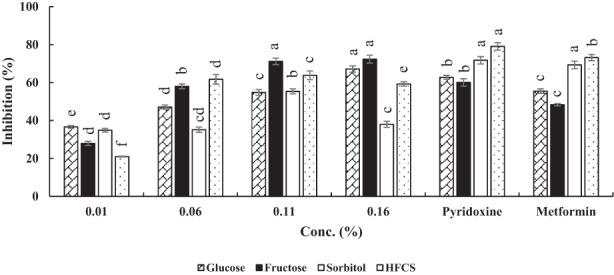
Antiglycation activities of whey protein hydrolysates in four model systems. Results are represented as mean ± SD (*n* = 3). Different letters on the columns indicate significant differences (*p* < .05). Concentration of metformin and pyridoxine was 1.0 mM.

As shown in Figure 3, WHP antiglycation activity in BSA‐Glucose system was less than 40% at 0.01%. By increasing the concentration up to 0.16%, percent of inhibition increased to 70%. Inhibitory effect of WHP was more than both standards.

In the BSA‐Fructose model system, inhibitory activity of WPH initiated from 27.95% and reached ~72% and then remained constant up to 0.16%. WPH antiglycation activity was better than two synthetic inhibitors (Figure 3).

In the BSA‐Sorbitol system, WPH showed a similar behavior with the BSA‐Glucose (at 0.01%). As observed from Figure 3, by increasing the WPH concentration, percent of inhibition did not show a specified trend. Pyridoxine and metformin had higher antiglycation activity than WPH. Lower degree of hydrolysis in whey proteins resulted in less availability of functional groups and consequently lower anti‐AGEs activity compared with two standards.

Finally, in BSA‐HFCS model, the percent of inhibition at 0.01% was exactly 20%. Afterward, by increasing the concentration, the antiglycation activity significantly increased and reached 59.0% (*p* < .05). As seen from Figure 3, WPH could not compete with two synthetic inhibitors.

By comparing the four examined systems and three protein hydrolysates, it is clear that in addition to peptide's concentrations, type of sugar is as important as other factors and should be taken into close consideration. Some sugars are less prone to the Maillard reaction and some are highly likely to participate, thoroughly the same as different types of amino acids (Xie et al., 2023). In the BSA‐Fructose system, hydrolysates had the lowest inhibitory activity. Nevertheless, it was found that the hydrolysates had highest activity in the BSA‐Glucose system. It seems that glucose has the lowest tendency to take part in the glycation (Kuerban et al., 2017; Kuerban, Al‐Gharafi, et al., 2020). The inhibitory activity of hydrolysates in the BSA‐HFCS system was more or less between the two BSA‐Fructose and BSA‐Glucose systems. As mentioned in Section 2.1, the fructose‐to‐glucose ratio of HFCS was 55: 45, so peptides inhibitory activity in the mentioned model was higher than the BSA‐Fructose and lower than the BSA‐Glucose system.

All three studied hydrolysates did not show a specified trend in the BSA‐Sorbitol system and results had some fluctuations. It seems, as reported by Deo et al. (2020), the structure of sugar alcohols, which are similar to reducing sugars, may contribute to the formation of AGEs and their reactive intermediates in both exogenous and endogenous models through autoxidation.

Protein hydrolysates are thought to suppress glycation and its development by different ways. Initially, peptides may act as a protecting agent for BSA and therefore, prevent it from participating in the Maillard reaction (Kuerban, Al‐Malki, et al., 2020; Sarmah & Roy, 2021). Under such circumstance, BSA's secondary structure would be protected and its intramolecular forces would not be broken and/or its structure will not be unfolded (Deng, Wang, Zhang, Xie, & Huang, 2023). It is important to note that BSA structure may become porous or loose throughout the Maillard reaction, which happens mainly at the early stages of the reaction and is due to thermal protein denaturation (Ke & Li, 2023; Zhu et al., 2022).

In addition, peptides are believed to have high antioxidant activity because of peptides functional amino groups. Therefore, they might interfere in the production of Schiff base and reduce Amadori products formation's rate (Kani et al., 2019). Possessing antioxidant properties, peptides are believed to scavenge free radicals, which act as greatly reactive intermediates, and therefore, peptides will suppress chain reaction and hinder protein structure variation (Jia et al., 2023). Therefore, antioxidant properties are generally accompanied by free radical trapping and preventing protein glycoxidation (Deng, Wang, Zhang, Zhang, et al., 2023).

Another mechanism for peptide's antiglycation activity is capturing precursors and therefore hindering glycation progress (Song et al., 2021). Since different precursors can originate different AGEs, namely, vesperlysine derived from glyoxal or argpyrimidine derived from methylglyoxal, to name but a few, peptides might own various capabilities in alleviating AGE precursor's generation (Chirinos et al., 2022).

As a result, peptides are highly capable of trapping reactive carbonyl and dicarbonyl groups and subsequently suppress the development of AGEs formation.

### 3.4 Effect of amino acids profile of protein hydrolysates on the antiglycation activity

As shown in Table 1, cysteine content in MPH, FPH, and WPH were 0.48, 0.46, and 2.42 g/100 g of hydrolysate, respectively. Cysteine contains thiol groups that have remarkable inhibitory effects on the Maillard reaction by interacting with dicarbonyl of reducing sugar, scavenging reactive oxygen species (ROS), and trapping electrophilic compounds (Mazumder et al., 2019; Sassetti et al., 2021). Tyrosine is another effective amino acid in the glycation reaction. This amino acid has hydroxyl group, and therefore, it can interact with active carbonyl and dicarbonyl groups by transferring electrons to free radicals and preventing the progress of reaction (Przybylski et al., 2016; Sarmah & Roy, 2021). Tyrosine contents of MPH, FPH, and WPH were 3.01, 5.32, and 3.46 g/100 g of hydrolysate, respectively.

By considering the probable relationship between hydrolysate's antioxidant and antiglycation activity, it can be concluded that hydrophobic amino acids such as valine, isoleucine play a key role in the antiglycation activity of hydrolysates. Because they have good antioxidant activities and as previously referred, there is a close correlation between antioxidant and inhibitory effect on protein glycoxidation (Karimi et al., 2020; Nirmal et al., 2022; Sarteshnizi et al., 2021). The total content of hydrophobic amino acids of MPH, FPH, and WPH were 30.27, 40.43, and 38.59 g/100 g of hydrolysate, respectively.

Taking every possible relationship between the type of amino acids and the inhibitory effect into account, it is concluded that the amount of effective amino acids of FPH was noticeably more than other two hydrolysates and that is why it showed the highest inhibitory levels.

Contrariwise, although the amount of effective amino acids of WPH was far more than that for MPH, the former showed lower inhibitory activity compared with the latter. This is because of the complexity of the protein matrix, molecular weight, different protein sources, composition, and sequence of amino acids affecting the interactions with sugars (Habinshuti et al., 2022; Xie et al., 2023). Thus, it requires more significant investigation to be thoroughly clarified.

## 4 CONCLUSION

Glycation is initiated by the reaction of reducing sugars with active amino groups of proteins (e.g., lysine) and resulted in AGE formation during some complicated stages. In addition, since AGEs could cause intermolecular protein cross‐linking, which if not prevented will alter or impair both biological and mechanical properties of proteins. In this study, effect of four sugar type and three protein hydrolysates as natural inhibitor of AGEs was investigated. It is obvious that these three studied hydrolysates were able to strongly inhibit AGEs production and aggregation at lower concentrations (Table 2). In addition, our results indicated that antiglycation activities of MPH, FPH, and WPH were higher than two chemical inhibitors such as pyridoxine and metformin. FPH had the strongest inhibitory effect on AGEs, whereas MPH and WPH had the second and third highest preventive influence. Hence, all studied hydrolysates can be suggested as potential functional ingredients for the control of AGEs in food processing.

**TABLE 2:** Comparison of antiglycation activities of the three studied hydrolysates with some recent reports.

Model system(s)	Inhibitor(s)	Function	References
BSA‐Glucose	Ten spices and plant extracts (3% v/v)	Antiglycation activities of allspice, thyme, green pepper, and black pepper were 60%, 45%, 40%, and 30%, respectively	Favre et al. (2022)
Cookies	Olive oil mill wastewater polyphenols (0.05%, 0.1%, 0.2%)	A 80% reduction in AGE precursor's formation was observed	Troise et al. (2020)
BSA‐Glucose, BSA‐Fructose	Red and brown *Lens culinaris* protein hydrolysates (0.5%)	The highest inhibitory effect belonged to red type in BSA‐Fructose (62.3%) model	Kuerban, Al‐Malki, et al. (2020)
BSA‐Glucose, BSA‐Fructose	*Vicia faba* peptides 0.5%	The highest and the lowest inhibition percent was for BSA‐Fructose (49.51%) and BSA‐Glucose (37.04%), respectively	Kuerban, Al‐Gharafi, et al. (2020)
BSA‐Glucose	Green pepper extract (3% v/v)	The best antiglycation activity was 72.8%	Favre et al. (2020)
BSA‐Glucose	High antioxidant‐leveled spices	Antiglycation activities of allspice, cloves, oregano, and star anise were 90%, 88%, 87%, and 81%, respectively	Starowicz and Zieliński (2019)
BSA‐Glucose	Synthetic inhibitors such as nimesulide, piroxicam, colchicine, meloxicam (1000 μM)	The best inhibitors were nimesulide, piroxicam, and colchicine with 86.24%, 77.37%, and 70.89%, respectively	Rasheed et al. (2018)
BSA‐Glucose BSA‐Fructose BSA‐Sorbitol BSA‐HFCS	Maize, Fish, and Whey protein hydrolysates with four selected concentrations between 0.01% and 0.16%	The best inhibitory activity of FPH, MPH, and WPH was ~99.0 (in BSA‐Glucose system), 90.1 (in BSA‐Sorbitol system), and 72.0% (in BSA‐Fructose system), respectively	This work

## CONFLICT OF INTEREST STATEMENT

There is no conflict of interest in this paper.

## ETHICAL APPROVAL

On behalf of all coauthors, I, Dr. Mohsen Barzegar, declare that this article has not been published in or is not under consideration for publication elsewhere. All authors were actively involved in the work leading to the manuscript and will hold themselves jointly and individually responsible for its content. Human or animal testing is unnecessary in our study.

## Data Availability

The data that support the findings of this study are available from the corresponding author upon reasonable request.
